# Neonatal intrahepatic cholestasis caused by citrin deficiency: prevalence and *SLC25A13* mutations among thai infants

**DOI:** 10.1186/1471-230X-12-141

**Published:** 2012-10-15

**Authors:** Suporn Treepongkaruna, Suttiruk Jitraruch, Porawee Kodcharin, Dussadee Charoenpipop, Pim Suwannarat, Paneeya Pienvichit, Keiko Kobayashi, Duangrurdee Wattanasirichaigoon

**Affiliations:** 1 Department of Pediatrics, Faculty of Medicine Ramathibodi Hospital, Mahidol University, Bangkok, 10400, Thailand; 2 Office of Research Academic and Innovation, Faculty of Medicine Ramathibodi Hospital, Mahidol University, Bangkok, 10400, Thailand; 3 Department of Molecular Metabolism and Biochemical Genetics, Kagoshima University Graduate School of Medical and Dental Sciences, Kagoshima, Japan

**Keywords:** AGC2 deficiency, Cholestatic jaundice, Idiopathic neonatal hepatitis, Infantile cholestasis, NICCD, Prevalence

## Abstract

**Background:**

The most common causes of cholestatic jaundice are biliary atresia and idiopathic neonatal hepatitis (INH). Specific disorders underlying INH, such as various infectious and metabolic causes, including neonatal intrahepatic cholestasis caused by citrin deficiency (NICCD) especially, in East Asian populations are increasingly being identified. Since most NICCD infants recovered from liver disease by 1 year of age, they often are misdiagnosed with INH, leading to difficulty in determining the true prevalence of NICCD. Mutation(s) of human *SLC25A13* gene encoding a mitochondrial aspartate/glutamate carrier isoform 2 (AGC2), can lead to AGC2 deficiency, resulting in NICCD and an adult-onset fatal disease namely citrullinemia type II (CTLN2). To study the prevalence of NICCD and *SLC25A13* mutations in Thai infants, and to compare manifestations of NICCD and non-NICCD, infants with idiopathic cholestatic jaundice or INH were enrolled. Clinical and biochemical data were reviewed. Urine organic acid and plasma amino acids profiles were analyzed. PCR-sequencing of all 18 exons of *SLC25A13* and gap PCR for the mutations IVS16ins3kb and Ex16+74_IVS17-32del516 were performed. mRNA were analyzed in selected cases with possible splicing error.

**Results:**

Five out of 39 (12.8%) unrelated infants enrolled in the study were found to have NICCD, of which three had homozygous 851del4 (GTATdel) and two compound heterozygous 851del4/IVS16ins3kb and 851del4/1638ins23, respectively. Two missense mutations (p.M1? and p.R605Q) of unknown functional significance were identified. At the initial presentation, NICCD patients had higher levels of alkaline phosphatase (ALP) and alpha-fetoprotein (AFP) and lower level of alanine aminotransferase (ALT) than those in non-NICCD patients (*p*< 0.05). NICCD patients showed higher citrulline level and threonine/serine ratio than non-NICCD infants (*p*< 0.05). Fatty liver was found in 2 NICCD patients. Jaundice resolved in all NICCD and in 87.5% of non-NICCD infants at the median age of 9.5 and 4.0 months, respectively.

**Conclusion:**

NICCD should be considered in infants with idiopathic cholestasis. The preliminary estimated prevalence of NICCD was calculated to be 1/48,228 with carrier rate of 1/110 among Thai infants. However, this number may be underestimated and required further analysis with mutation screening in larger control population to establish the true prevalence of NICCD and AGC2 deficiency.

## Background

Cholestatic jaundice affects approximately 1 in every 2,500 infants world-wide [1,2]. The most common causes are biliary atresia and idiopathic neonatal hepatitis (INH). An increase in specific disorders underlying INH are being identified, which include various infectious and metabolic causes such as tyrosinemia, alpha-1 antitrypsin deficiency, and galactosemia [3]. INH could be caused by unidentified metabolic disorders.

Deficiency of AGC2, liver-type mitochondrial aspartate-glutamate carrier (AGC) [4], is an autosomal recessive disorder caused by mutations of the *SLC25A13* gene (7q21.3) [5]. It results in two distinct phenotypes: adult-onset type II citrullinemia (CTLN2; OMIM 603471), and neonatal intrahepatic cholestasis caused by AGC2 deficiency (NICCD; OMIM 605814) [6]. CTLN2 is characterized by recurrent episodes of altered mental status, hyperammonemia and coma which can occur at any age but usually in adulthood [5,7,8]. NICCD patients present in the first few months of life with milder symptoms characterized by intrahepatic cholestasis, diffuse fatty liver, parenchymal cellular infiltration associated with hepatic fibrosis, hypoalbuminemia, coagulopathy, liver dysfunction with or without hypoglycemia, galactosuria, multiple aminoacidemia including elevated citrulline, arginine, threonine, methionine, phenylalanine, and tyrosine concentrations [8-12].

NICCD was first described by Ohura *et al.* in 2001 [13]. Increasing numbers of NICCD patients have been reported with the majority from Japanese and East Asian populations, including Taiwanese [14,15], Korean [16] and Chinese [12,17]. A handful of patients with NICCD have been identified in Arabic, Pakistani, Caucasian descendants, suggesting a panethnic disease [16,18-20]. Since most NICCD infants recovered from liver disease by 1 year of age, they often are misdiagnosed with INH, leading to difficulty in determining the true prevalence of NICCD [10]. Because clinical manifestations and biochemical findings are nonspecific for NICCD, DNA analysis or Western blot analysis of AGC2 protein in lymphocytes is the most reliable diagnostic tool [21]. The objectives were to study the prevalence of NICCD in Thai infants with idiopathic cholestasis, mutation spectrum of *SLC25A13* in Thai NICCD, and comparison of clinical manifestations and blood chemistry between NICCD and non-NICCD infants.

## Results

Thirty-nine unrelated infants with idiopathic cholestasis or INH participated in the study, 18 from cohort-A (12 males and 6 females) and 21 from cohort-B (17 males and 4 females), making a male/female ratio 3:1. Median age at onset of the jaundice was 1 month (range 0.5-5.0) in both cohorts. The median age at enrollment was 60.5 months (range 12.0-131.0) in cohort-A, and 3.0 months (range 1.0-16.0) in cohort-B. At the time of enrollment, in cohort-A, jaundice was resolved in all but one patient, whereas all patients in cohort-B presented with jaundice. Five male patients (1 from cohort-A and 4 from cohort-B) were confirmed to have NICCD.

### Mutation data

**Figure 1:**
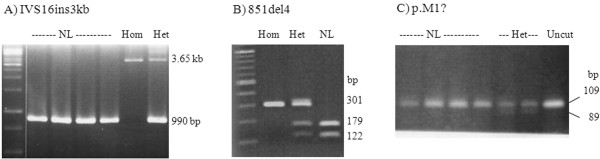
**Agraose gel electrophoresis showing IVS16ins3kb (XIX), Mutations 851del4 (I), and p.M1? variant.A**: 1.5% gel revealing 3.65 kb fragment representing the IVS16ins3kb (XIX) and the 990bp fragment representing normal allele.; **B**: 1.5% gel showing exon 9-PCR-*Hpy*CH4IV restriction digest. The 851del4 mutation abolishes the restriction site, whereas the normal allele was cut, yielding 179 and 122bp fragments.; **C**: 2.5% gel showing 109bp-product of PCR using primers E1F/E1_1MT-R. The p.M1? allele produces an *Eag*I restriction site, yielding 89 and 20bp-fragments.

Genotypes in the NICCD cases were homozygous 851del4 or GTATdel (mutation [I]) in Patients 1, 2, and 5, 851del4/IVS16ins3kb (or mutation [XIX]) in Patient 3 (Figure 1A), and 851del4/c.1638-1660dup (or mutation [III]: 1638ins23) in Patient 4. There were two suspected NICCD cases with genotype p.M1?/wt in Patient 6 and R605Q/wt in Patient 7 (both from cohort-A). None of the patients had mutation [XX] (or Ex16+74_IVS17-32del516). Two reported single nucleotide polymorphisms (SNPs) including IVS4+6A>G (rs6957975) and L398L (rs2301629); and 2 newly identified SNPs, IVS4-52 A>G and IVS17-12C>A were discovered (Table 1).

**Table 1:** Frequency of genetic variations screened or identified in this study

**Exon/intron**	**Genetic variation**	**Conventional name**	**Protein change**	**Pathogenicity**	**Cholestatic infants (AF)**	**Controls (AF)**	**Reference**
Exon 1	2T>C	NA	p.M1?	unknown	1/39 (het: Pt-6) (0.013)	3/100 (0.015)	present study
Intron 4	IVS4+6A>G	NA	none	benign	AA 17/39; AG 18/39; GG 4/39 (AF: A 0.67; G 0.33	NA	rs6957975; ss28503034
Intron 4	IVS4-52 A>G	NA	none	benign	1/39 (het: Pt-8) (0.013)	NA	present study
Exon 9	c.851delGTAT	I	p.M285fsX286	pathogenic	5/39 (hom: Pt-1,2,5 and het: Pt-3,4) (0.103)	0/100	Kobayashi 1999 [5]
Exon 12	c.1194A>G	NA	p.L398L	benign	AA 9/39; AG 12/39; GG 18/39 (AF:A 0.385; G 0.615)	NA	rs2301629
Exon 17	c.1814G>A	NA	p.R605Q	unknown	1/39 (het: Pt-7) (0.013)	0/100	present study
Exon 16	c.1638-1660dup (1638ins23)	III	p.A554fsX570	pathogenic	1/39 (het: Pt-4) (0.013)	0/100	Kobayashi 1999 [5]
Intron 16	IVS16ins3kb	XIX	p.A584fsX585	pathogenic	1/39 (het: Pt-3) (0.013)	0/100	Tabata 2008 [20]
Intron 17	IVS17-12 C>A	NA	none	benign	1/39 (het: Pt-9) (0.013)	NA	present study

*AF* allele frequency, Cholestatic infants, idiopathic infantile cholestasis individuals, *hom* homozygous, *he*t heterozygous, *NA* not applicable/available, *Pt* patient. GenBank reference sequences were NT_079595 and NM_014251.2.

After an identification of 851del4 in Patients 1-5, we established a screening method using PCR with primer SLC25A13_E9 forward and reverse (for all primers, see Additional file 1) followed by *Hpy*CH4IV (A′CGT) restriction digestion (Figure 1B). This test was used for simple and rapid screening of the 851del4 in the control specimens and also as a second method of confirming the deletion in the patients.

The p.M1? variant does not alter restriction site, therefore, a reverse primer E1_M1T-R was designed and used with forward primer E1-F to yield PCR product containing the mutated site and to create an artificial *Eag*I restriction site (C′GGCCG) in the p.M1? allele (Figure 1C). This test was used to screen the 100 controls.

Sequencing of cDNA from an 851del4-homozygous patient and the p.R605Q-heterozygous individual revealed correct splicing of the exons involved (data not shown). None of the controls were found to have 851del4, IVS16ins3kb, 1638ins23, and the p.R605Q variant (creating a *Bsr*DI restriction site). The p.M1? was present in only heterozygous state in 3 controls (Table 1).

### Comparison of clinical and laboratory findings between NICCD and non-NICCD infants

The major presentations of five definite NICCD patients (Patients 1-5) were cholestatic jaundice and hepatomegaly. Median age at onset of cholestatic jaundice was 0.5 months (range 0.1-4.0) in NICCD patients and 1.0 month (range 0.5-5.0) in non-NICCD patients (Patients 8-39). Splenomegaly was initially found in 2 NICCD (40%) and 11 non-NICCD patients (34.4%). At presentation, NICCD patients had significantly higher alkaline phosphatase (ALP), lower ALT and higher alpha-fetoprotein (AFP) than in non-NICCD infants (Table 2). Coagulopathy, galactosuria and hypoglycemia were remarkably observed among NICCD patients but not in the non-NICCD infants. Serum AFP levels were not available in suspected NICCD cases. Plasma amino acid (PAA) profiles were only available from four NICCD patients (from cohort-B) and 18 non-NICCD patients which showed elevated citrulline and elevated threonine/serine ratio in NICCD patients compared to those in non-NICCD patients (*p*<0.05) (Table 3).

**Table 2:** Liver functions and laboratory data at the first presentation of definite NICCD, and non-NICCD patients

**Parameters**	**Definite NICCD (n=5) median (range)**	**Non-NICCD (n=32) median (range)**	**Normal value**	***P*-value**^**a**^
TB (mg/dL)	7.1 (4.7-9.8)	7.6 (3.5-49.9)	0.2-1.0	0.61
DB (mg/dL)	3.8 (2.6-6.9)	5.9 (1.8-40.1)	0.0-0.7	0.18
ALP (U/L)	1016 (665-3014)	540 (256-1935)	185-555	0.01
GGT (U/L)	160 (127-289)	188 (35-936)	12-123^b^	0.86
AST (U/L)	144 (62-268)	173 (63-942)	15-37	0.48
ALT (U/L)	61 (49-127)	135 (28-525)	30-65	0.03
AST/ALT ratio	2.1 (1.1-3.3)	1.3 (0.6-5.2)	-	0.07
TP (g/L)	51 (38-61)	54 (34-75)	64-82	0.42
Albumin (g/L)	33 (25-37)	35 (17-38)	34-50	0.33
AFP (ng/mL)	60500 (38930 – 60500) (n=4)	22678 (30-60500) (n=9)	88±87^c^	0.01
Hypoglycemia^d^	2 (40%)	1 (3.1%)	-	0.01
Galactosuria	3/4 (75%)	0		-
Prolonged PT^e^	2/3 (67%)	6/24 (25%)	-	0.14

*TB* total bilirubin, *DB* direct bilirubin, *ALP* alkaline phosphatase, *GGT* γ-glutamyltransferase, *AST* aspartate aminotransferase, *ALT* alanine aminotransferase, *TP* total protein, *AFP* alpha-fetoprotein, *PT* prothrombin time, *NA* not available.

^a^ Only definite NICCD and non-NICCC groups were compared, and statistical significance if *P*-value <0.05.

^b^ Normal values for children 1-2 months, 12-123; 2-4 months, 8-90; 4 months-10 years, 5-32 U/L.

^c^ Normal values for children at 3 months, 88±87; 5 months, 46.5±19; 6 months, 12.5±9.8; 8-12 months, 8.5±5.5 ng/mL.

^d^ Hypoglycemia defined as blood glucose <2.5 mmol/L or 45 mg/dL.

^e^ Prolonged PT defined as INR>1.2.

**Table 3:** Plasma amino acids (PAA) of NICCD and non-NICCD infants with jaundice at enrollment

**PAA**	**NICCD (n=4) median (range)**	**Non-NICCD (n=18) median (range)**	**Normal value**	***P*-value**^**b**^
Citrulline (umol/L)	60 (36-152)	17 (8-42)	3-35	0.004
Methionine (umol/L)	141 (32-243)	37 (11-702)	9-42	0.23
Phenylalanine (umol/L)	38 (26-40)	45 (11-291)	31-75	0.19
Threonine (umol/L)	181 (139-326)	140 (42-339)	24-174	0.11
Arginine (umol/L)	63 (50-135)	41 (12-92)	12-133	0.16
Tyrosine (umol/L)	121 (33-166)	74 (18-557)	22-108	0.49
Threonine/serine ratio^a^	1.63 (1.37-2.10)	1.16 (0.56-2.30)	1.10 (0.88-1.19)	0.03
Fisher ratio (BCAA/AAA)	1.12 (0.67-2.47)	2.23 (0.01-3.42)	3.42±0.33	0.13

*BCAA* branched chain amino acids (valine + isoleucine + leucine), *AAA* aromatic amino acids (phenylalanine + tyrosine).

^a^ median and 25-75% range of threonine/serine ratio in NICCD 2.29 (1.69-2.81) were as described by Kobayashi K and Saheki T (GeneReviews) [8].

^b^ Only definite NICCD and non-NICCC groups were compared, and statistical significance if *P*-value <0.05.

Liver histology was available in 3 NICCD and 15 non-NICCD infants. Cholestasis and fatty changes were found in two NICCD patients (Patients 3 and 4). In addition, histologic features of cirrhosis were demonstrated in Patient-4. Patient-2 had marked cellular swelling, disarray of hepatic cords, cholestasis, multinucleated giant cell transformation, and decreased interlobular bile ducts. The major histological findings in non-NICCD infants were multinucleated giant cell transformation and features compatible with INH (n=9), paucity of interlobular bile duct (n=4), fatty changes (n=1; Patient 10) and severe periportal fibrosis (n=1). The liver histologic findings of the 2 suspected NICCD patients were compatible with INH.

The management of all the patients was directed toward treating the consequence of cholestasis, consisting of non-lactose and medium chain triglycerides formula and supplementation with fat-soluble vitamins. Jaundice resolved in all NICCD patients at the median age of 6.6 months (range 5-11) and liver biochemistry became normal in 4/5 NICCD patients (except patient 4) at the median age of 9.5 months (range 5-10). Patient 4 had the most severe manifestation in the NICCD group in this study. He had jaundice started at 1 month old, developmental delay due to unrecognized hypoglycemia till the age of 8 months when he was referred to our department. After 4 months of treatment, jaundice and hypoglycemia resolved and liver chemistry became near normal. At the time of this report, he is 38 month-old with appropriate growth and psychomotor development.

Jaundice resolved in the two suspected NICCD cases, at the age of 5 and 9 months, respectively. Among the non-NICCD infants, 87.5% (28/32) had jaundice resolved at median age of 4 months (range 2-24). The remainder had progressive jaundice leading to cirrhosis; one patient underwent liver transplantation and has been well, and another patient died of complications of end-staged liver disease.

## Discussion

In the present study, the 851del4 was accounting for 80% of the mutant alleles identified. We demonstrate 12.8% (5/39) prevalence of NICCD among Thai infants with idiopathic cholestatic jaundice/INH. Ko et al and Fu et al reported 6% (3/47) and 9.5% (38/400) prevalence of NICCD among Korean and Chinese infants with idiopathic cholestasis, respectively [22,23]. By employing the above prevalence of 12.8%, 40% of Thai infantile cholestatsis being have idiopathic cholestasis [24] (and our unpublished data), and panethnic incidence of cholestatic jaundice 1/2,500 infants (no specific data for Thai infants) [1,2], the incidence of NICCD among Thai infants is calculated to be 1 in 48,828. By reverse calculation based on Hardy-Weinberg equation, the frequency of *SLC25A13* mutation-carrier among Thai infants calculated from the incidence of NICCD is predicted to be 1 in 110 (q^2^=1/48,828; 2pq = 2x≅1x1/220). This number is lower than the frequency of *SLC25A13* mutations carrier among Chinese (1/63), Japanese (1/65), and Korean (1/108) populations, respectively [15,20,25], plausibly due to underestimation of the prevalence in the present study because not all infants with *SLC25A13* mutation(s) on both alleles manifest NICCD symptoms and that the carrier rates described in those East Asian populations were obtained through molecular genetic screening of control population which is more reliable method to provide epidemiologic data.

If p.M1? is considered pathologic allele and its carrier rate (3/100) by population analysis is taken into account with the carrier rate of the three mutations (851del4, IVS16ins3kb, and 1638ins23) obtained from the cholestatic infants group, it makes a carrier rate of 1 in 52 (4/210), giving a new estimated incidence of NICCD 1 in 11,025. Given no available data of p.M1? in other population to compare and the absence of functional prove of the p.M1?, it may be too premature to include the carrier rate of p.M1? for estimation of the incidence of NICCD in Thai population.

We tried to compare the genotype of the most severe case (Patient 4) in the present study to those severe NICCD cases published in the literature. Only four NICCD patients have been reported to have progressive liver disease requiring liver transplantation [9,26-28]. Genotypes of those cases were 851del4/IVS11+1G>A [9,27], 1638ins23/S225X [26], and genotype not specified in two cases; therefore, genotype-phenotype association could not be concluded.

Among over 50 mutations of *SLC25A13* described [25], the 851del4 (mutation I) and IVS11+1G>A (mutation II) are the most frequent mutations described among Japanese affected population, accounting for 70% of the mutant alleles, and 851del4 is the most frequent in Chinese [8,20]. The other common mutations were 1638ins23, S255X, and IVS6+5G>A [20]. There is no doubt about the pathogenicity of the mutations 851del4, IVS16ins3kb, and c.1638-1660dup because these mutations have been identified multiple times in East Asian affected population although, functional data is quite limited.

The pathogenicity of the novel p.M1? variant is uncertain. With the absence of the original initiation start codon, the first AUG codon is located at nt 71-73 leading to translation of a non functional short polypeptide of 21 amino acids and possible degradation of the mutant mRNA through a nonsense-mediated mRNA decay mechanism [29]. Whether or not this particular allele has deleterious effect on AGC2 function, it remains to be elucidated. The p.R605Q mutation occurs at a conserved amino acid position of *SLC25A13* (AGC2) and *SLC25A12* (aralar) across various species (AGC2: cow, chicken, mouse, rat, chimpanzee, horse; aralar: human, monkey, macaque, dog, mouse); although possibly be deleterious, its pathogenicity remains to be proven.

When comparing with the non-NICCD cases, the NICCD patients had significantly higher ALP and lower ALT levels, higher citrulline concentration and threonine/serine ratio, supporting previous studies [9,11,30]. The AST/ALT ratio seemed higher in NICCD group, but was not statistically significant. Failure to thrive was a presenting feature in 37-50% of NICCD patients, but not found in our cohort [11,12]. NICCD cannot be excluded on the basis of normal PAA profiles, as evidenced in Patients-3. Liver histology was not always diagnostic for NICCD. The characteristic histology of NICCD including cholestatsis and fatty change [10,11,31,32] was found in two cases in this study. Bile duct paucity, an uncommon finding in NICCD which has been demonstrated in one report [31] was found in one patient (Patient 2). His clinical manifestations were moderately severe with cholestatic jaundice, coagulopathy, galactosuria and jaundice resolved at 6 months of age. Fatty liver was found in a non-NICCD patient (Patient 10) who had jaundice since 1 month and the jaundice resolved by 3 months without identified other metabolic liver diseases. Another non-NICCD case, Patient 11, had markedly high ALP and AFP (1935 and >60,500 respectively, shown in Table 2). Her jaundice started at 1 month and resolved at 6 months of age, and her liver histology was the feature of INH. Of note, Patients 10 and Patient 11 could have NICCD but with unidentifiable mutation(s).

There were limitations of the present study. Firstly, using the panethnic incidence of infantile cholestasis for the estimation of the prevalence of NICCD among Thai infants raises concern on the precision of the predicted prevalence. It would be more reliable to use prevalence of infantile cholestasis among Thai population; however, such data does not exist. Secondly, the prevalence of NICCD obtained by calculation from manifesting infants may be underestimated because AGC2 deficiency is a disease with incomplete penetrance. Thirdly, large deletion and/or insertion could be missed by PCR-direct sequencing. Fourthly, the lack of functional analysis to support the pathologic significance of novel *SLC25A13* variants identified in the present study leads to inconclusive data of some patients (Patients 6 and 7). Existing system for functional analysis of AGC2 protein is complicated and difficult to conduct [33]. New method of functional analyses of AGC2 mutation is needed to provide more insight into *SLC25A13* variants.

## Conclusion

NICCD has been underestimated and should be considered in infants with idiopathic cholestatic jaundice, especially when associated with markedly high ALP and AFP, fatty liver and elevated plasma citrulline. It is impossible to establish the true incidence of NICCD and AGC2 deficiency among Thai infants by the method used in the present study. Molecular genetic analysis of general population and functional analysis to indicate the disease association with the variants is required to answer that question. Development of alternative and simple system for functional testing for AGC2 variants is necessary for better understanding pathogenic mechanism of AGC2 genetic variants, especially for those newly identified.

## Material and methods

### Patients and clinical analysis

Infants with idiopathic cholestasis or idiopathic neonatal hepatitis were recruited. Those diagnosed during January 1996 - September 2006 were assigned to cohort-A (retrospective cohort), and those diagnosed during October 2006 - February 2010 to cohort-B (prospective cohort). Idiopathic cholestasis was defined as serum total bilirubin (TB) exceeding 5 mg/dL and direct bilirubin (DB) level > 20% of TB or DB > 1 mg/dL if TB was < 5 mg/dL, and without an identifiable cause. Written informed consents were obtained from each parent, following the approval of Ramathibodi Institutional Review Board. Clinical data were reviewed. Routine investigations for infantile cholestasis included viral studies, abdominal ultrasonography, hepatobiliary scintigraphy and metabolic screening (urine reducing substance). Biliary atresia and extrahepatic obstruction were excluded in all cases. Liver biopsy was performed if the diagnosis remained inconclusive. Urine organic acids and plasma amino acid (PAA) were analyzed in the patients from cohort-A who still had jaundice at the time of enrollment, and all patients from cohort-B. Fischer and threonine/serine ratio were calculated (Fischer ratio = branched-chain amino acids valine + leucine + isoleucine/aromatic amino acids tyrosine + phenylalanine). Specific investigations for progressive familial intrahepatic cholestasis (PFIC), Alagille syndrome and specific metabolic liver diseases were performed in selected cases. Investigations for alpha-1 antitrypsin deficiency were not routinely performed since a previous study showed that this disorder is unlikely to be a cause of liver diseases among Thai children [34].

### Genetic analysis

Genomic DNA (gDNA) was prepared from peripheral blood lymphocytes by phenol-chloroform extraction. PRIMER 3 was used for primer design (http://www.Frodo.wi.mit.edu/cgi-bin/primer3). GenBank reference sequences were NT_079595 and NM_014251.2 (AGC2 isoform 2). Sequencing was performed on an ABI 3100 DNA sequencer after purification with QIAquick PCR purification kits (QAIGEN®; California, USA). Long-range PCR was performed according to the manufacturer’s instructions (Long PCR Enzyme Mix; Fermentas Life Sciences, California, USA). mRNA transcripts were isolated from peripheral blood with the QIAamp RNA Blood Mini Kit (QIAGEN^TM^), then reverse transcribed into single-stranded complementary DNA (cDNA) using Superscript III Reverse Transcriptase (Invitrogen). Primer sequences for gDNA and cDNA amplification are available as supplementary data at the journal website.

PCR-sequencing of all 18 exons of the *SLC25A13* gene of genomic DNA from all the infants and of cDNA from selected cases was performed. To detect both 3kb insertion of intron 16 (mutation [XIX] or IVS16ins3kb) and 516bp deletion of exon 16 connecting intron 17 ([XX] or Ex16+74_IVS17-32del516), gap PCR was performed by using long-range PCR and primers Ex16F and Ex18-3′R, as previously described [20]. Positive controls for the insertion were kindly provided by Prof. Kobayashi from Kagoshima University. Once a mutation(s) was identified, genetic analysis of family members was performed and PCR-restriction digest with appropriate endonuclease restriction enzymes was carried out in 100 healthy controls (regular blood donors). The IVS16ins3kb was also screened in the 100 controls. cDNA analysis was performed in selected case with possible splicing error. Primer sequences and detailed molecular method are available as an Additional file 1. Nomenclature of newly identified *SLC25A13* variants is assigned following the guidelines of the Human Genome Variation Society (http://www.hgvs.org/mutnomen) [35].

Infants with pathogenic *SLC25A13* mutation(s) identified on both alleles were considered definite case of NICCD while those with pathogenic/unclear significant mutation identified on only one allele were considered suspected NICCD, and those known as nonpathogenic/silent or unidentifiable mutation were considered non-NICCD cases.

### Statistical analysis

Data were analyzed using SPSS (version 13.0, SPSS Inc, Chicago, Illinois, USA). Mann–Whitney *U* test and Fisher's Exact test were used to compare continuous variables and dichotomous variables, respectively. A *P*-value of <0.05 was considered statistically significant.

## Competing interests

The authors declare that they have no competing interest.

## Author’s contributions

ST, SJ, PP, collected and analyzed clinical data, and wrote/edited the manuscript. DW, PS, SJ designed laboratory work and wrote/edited the manuscript. PK and DC performed laboratory work and edited the manuscript. KK advised the study design and editing the manuscript. All authors read and approved the final manuscript.

## Pre-publication history

The pre-publication history for this paper can be accessed here:

http://www.biomedcentral.com/1471-230X/12/141/prepub

## Supplementary Material

Additional file 1 Primer sequence and molecular method. Click here for file
