# Supplementary material for: Neonatal intrahepatic cholestasis caused by citrin deficiency: prevalence and SLC25A13 mutations among thai infants
Source: BMC Gastroenterol. 2012 Oct 15;12:141. doi: 10.1186/1471-230X-12-141 (PMC3483206; doi:10.1186/1471-230X-12-141)
Supplement: Additional file 1 — Primer sequence and molecular method. [file 1471-230X-12-141-S1.pdf]

Additional file_1 Table. Genomic and cDNA Primers for SLC15A13

Primer names	Forward primer (5'-3')	Reverse primer (5'-3')	AT (^{o}C)	fragment size (bp)
genomicDNA primers				
SLC25A13_E1	GCCGGGACTAGAAGTGAGC	accaacccagacacgtgag	60	190
SLC25A13_E2	cactggggcaacatggtatt	gctgacactttgggactttttc	56	310
SLC25A13_E3	gaactgttgggagataatggtc	tggtcctaagagatgggaagg	66	407
SLC25A13_E4	ccctcagtgtatgtttgtatatgct	gaaaatgctcacacaagtccac	60	250
SLC25A13_E5	ctgattccttgagggttcttt	gcctcccaaagtgctaggat	60	389
SLC25A13_E6	taggcagatgagggcttgtt	cccactaaaactagccaaaacac	56	451
SLC25A13_E7	tttcttcttgtacatgcatttttga	ctagttgccttcttcaccctaa	56	332
SLC25A13_E8	tggatgcaagaactgcaaa	gccctcctcctaacctcctt	56	329
SLC25A13_E9	aagccaaactgaaggctatactg	cagataccaatgccgcaaag	56	301
SLC25A13_E10	catggatttagaacccaatgag	aactggcattgggaaagact	56	316
SLC25A13_E11	cacatttcttttctcaatcttgtcc	tccattttaacgcagtcttgc	62	362
SLC25A13_E12	gcagaaaattgcagcagaga	gcacctctgagaaaacaaacc	57	404
SLC25A13_E13	ttgtgcaatttatgattactccaa	atggttcgcctgtctaggaa	64	371
SLC25A13_E14	tgggacaagggctgaaacta	tctgcagcttgggtagaaca	62	404
SLC25A13_E15	tgttcccctttcaatgtgtg	tagcatgcagctagggaagg	64	306
SLC25A13_E16	ccagcagttcaaagcacagt	ggggtgaggatcgaaataca	57	353
SLC25A13_E17	acagcggagtgatagactgc	ttccctacgacaacagagca	62	324
SLC25A13_E18	aggtcatttccaaggggaac	CCCAGGAGGGATGTTCTTTA	57	396
Ex16F[a]	gtatgcctgcagcatctttag		61	990
Ex18-3'R[a]		TGCTTCATTCCCAGGAGGGA		
E1_M1T-R[b]		CGCGGTTACCTTGGCGGC***G***G		
cDNA primers				
cSLC25A13_E7-E11	TCGCTCCTTAACAACATGGA	GCCAGTTGATCGTTGGTTCT	66	417
cSLC25A13_E6-E12	CCCCATGTCTTGACTCCTTT<br>(nt574-594)	ACTGTGGCAACAGACCTCTA<br>(nt1192-1173)	66	619

AT, annealing temperature. small letter for intronic sequence, and capital letter represents exonic sequence

Most primers are newly designed using program PRIMER 3; http://www.Frodo.wi.mit.edu/cgi-bin/primer3

[a] Ref: Tabata et al. J Hum Genet (2008) 53:534-545AT. [b] M1T-R is mismatched at nucleotide 6 downstream from the start codon, producing a G instead of a wild-type C four bases downstream from the mutation.
